# LcpB Is a Pyrophosphatase Responsible for Wall Teichoic Acid Synthesis and Virulence in *Staphylococcus aureus* Clinical Isolate ST59

**DOI:** 10.3389/fmicb.2021.788500

**Published:** 2021-12-16

**Authors:** Ting Pan, Jing Guan, Yujie Li, Baolin Sun

**Affiliations:** ^1^Department of Oncology, The First Affiliated Hospital of USTC, Division of Life Sciences and Medicine, University of Science and Technology of China, Hefei, Anhui, China; ^2^Department of Gastroenterology, The First Affiliated Hospital of Anhui Medical University, Hefei, China

**Keywords:** LCP family, pyrophosphatese, *Staphylococcus aureus*, virulence, wall teichoic acid synthesis, *agr* system

## Abstract

The community-associated methicillin-resistant *Staphylococcus aureus* (CA-MRSA) causes severe pandemics primarily consisting of skin and soft tissue infections. However, the underlying pathomechanisms of the bacterium are yet to fully understood. The present study identifies LcpB protein, which belongs to the LytR-A-Psr (LCP) family, is crucial for cell wall synthesis and virulence in *S. aureus*. The findings revealed that LcpB is a pyrophosphatase responsible for wall teichoic acid synthesis. The results also showed that LcpB regulates enzyme activity through specific key arginine sites in its LCP domain. Furthermore, knockout of *lcpB* in the CA-MRSA isolate ST59 resulted in enhanced hemolytic activity, enlarged of abscesses, and increased leukocyte infiltration. Meanwhile, we also found that LcpB regulates virulence in *agr*-independent manner and the key sites for pyrophosphatase of LcpB play critical roles in regulating the virulence. In addition, the results showed that the role of LcpB was different between methicillin-resistant *Staphylococcus aureus* (MRSA) and methicillin-sensitive *Staphylococcus aureus* (MSSA). This study therefore highlights the dual role of LcpB in cell wall synthesis and regulation of virulence. These insights on the underlying molecular mechanisms can thus guide the development of novel anti-infective strategies.

## Introduction

*Staphylococcus aureus* is a major human pathogen that can cause diseases ranging from minor skin infections to life-threatening osteomyelitis, sepsis, pneumonia, and toxic shock syndrome (Lowy, 1998). Some antibiotics such as penicillin, methicillin and vancomycin were introduced to treat *S. aureus* infection. However, *S. aureus* quickly adapted to the pressure of antibiotics and generated drug-resistant strains (Deleo and Chambers, 2009). Among them, methicillin-resistant (MRSA) strains pose a serious threat because of their rapid epidemic spread and enormous virulence potential, especially CA-MRSA strains (DeLeo et al., 2010). Multiple macromolecules are displayed on the surface of MRSA, including individual proteins, protein polymers, polysaccharides, and anionic polymers (e.g., teichoic acids) (Kawai et al., 2011). Wall teichoic acid (WTA) is a highly abundant modification of the cell wall and one of the most diverse surface determinants of *Staphylococcus aureus* (van Dalen et al., 2020). It is a polymer composed of ribitol-phosphate monomers which can be repeated up to 40 times (Covas et al., 2016). Poly-ribitol-phosphate is assembled upon a polyprenyl-pyrophosphoryl lipid carrier which is anchored to the C6-hydroxyl of N-acetylmuramic acid residue within the peptidoglycan strands through a phosphodiester bond (Brown et al., 2013; Mann et al., 2016). WTA contributes to a variety of processes in the bacterial metabolism including resistance to antibiotics, biofilm formation, cell division, especially virulence (Lee et al., 2016). Recent studies have shown that the *agr* system can enhance the infection of *S. aureus* by regulating WTA synthesis (Wanner et al., 2017). Therefore, it has attracted extensive attention as a target structure for novel anti-infective strategies and antibiotics (Hubscher et al., 2008; Pasquina et al., 2013; Wang et al., 2013; Maria et al., 2014; Lehar et al., 2015; Ling et al., 2015; Winstel et al., 2015; Siegel et al., 2019).

The LytR-CpsA-Psr (LCP) proteins are unique to Gram-positive bacteria and named for its LCP domain (Srisuknimit et al., 2017), but the function of LCP domain is still unclear. Bacterial genomes usually encode several (up to 11) LCP proteins, which have a common structure consisting of a short N-terminal cytoplasmic domain, a transmembrane region with 1–3 transmembrane helixes, and an extracellular region containing LCP domain (Srisuknimit et al., 2017). LCP proteins are considered to be a pyrophosphatase or phosphotransferase which is involved in the process of cell wall synthesis and mediate the attachment of capsular polysaccharide to peptidoglycan (Over et al., 2011; Chan et al., 2014; Harrison et al., 2016; Schaefer et al., 2017; Li F. K. K. et al., 2020). In addition, recent study has shown that the LCP protein plays an important role in the process of infecting the host (Li F. et al., 2020). As a target enzyme, inhibitors of LCP enzymes have the potential to manage a wide range of bacterial infections. In addition, the LCP family represents an attractive class of drug targets in which the soluble catalytic region is on the extracellular face of the cytosolic membrane and there are no mammalian orthologs (Srisuknimit et al., 2017).

In *S. aureus*, there are three LCP proteins: LcpA, LcpB, and LcpC (Huebscher et al., 2009; Chan et al., 2013). The existing researches mainly focus on the analysis of LcpA (Rossi et al., 2003; Huebscher et al., 2009; Li F. K. K. et al., 2020). LcpB is also involved in multiple physiological processes (Chan et al., 2013). However, the present understanding of LcpB and LCP domain is poor. Here, we focus here the role and the regulatory mechanism of LcpB in WTA synthesis and virulence in the CA-MRSA clinical isolate ST59. We have shown that *lcpB* deficiency slows down the growth, accelerates the autolysis and resulted in abnormal cell wall morphology. Combined with enzyme activity detection and WTA analysis, we have confirmed that LcpB is a pyrophosphatase and participates in the synthesis of WTA. Meanwhile, we identified the key arginine sites in LCP domain that affect its pyrophosphatase activity. Finally, using hemolytic activity detection and mouse subcutaneous abscess model, we found LcpB regulation was *agr*-independent and the key sites for pyrophosphatase of LcpB play critical roles in regulating the virulence. Finally, we demonstrated that the role of LcpB was strain specific.

## Materials and Methods

### Bacterial Strains, Plasmids, and Growth Conditions

The bacterial strains and plasmids used in this study are described in Supplementary Table 1. *Escherichia coli* were grown in Luria broth (LB) medium (Oxoid). *S. aureus* and its derivative strains were grown in tryptic soy broth (TSB) medium (BD) at 37°C with shaking at 220 rpm. When needed, appropriate antibiotics were used for plasmid selection and maintenance at the following concentrations: for *E. coli*, ampicillin at 150 μg/mL and kanamycin at 50 μg/mL; for *S. aureus*, chloramphenicol at 15 μg/mL. Constructed plasmids were purified from *E. coli Trans1*-T1 and transformed into *S. aureus* RN4220 as the initial recipient and then *S. aureus* strain ST59 by electroporation. The media were solidified with 1.5% (w/v) agar when required.

### DNA Manipulation

*S. aureus* genomic DNA was prepared by a standard protocol for Gram-positive bacteria. Plasmid DNA was extracted with a plasmid purification kit (Sangon Biotech) according to the manufacturer’s instructions. PrimeSTAR HS DNA polymerase (TaKaRa) and Phanta Max Super-Fidelity DNA polymerase (Vazyme) were used for PCR amplification, respectively. All plasmids transformed into the target *S. aureus* strain were first introduced into *S. aureus* strain RN4220 by electroporation at 2.5 kV for modification. The primers used in this study are listed in Supplementary Table 2.

### Construction of *Staphylococcus aureus* Mutant Strains

To construct the *lcpB* mutants, the upstream and downstream fragments of *lcpB* were amplified from *S. aureus* strain ST59 genomic DNA using the *lcpB*-up-F/lcpB-up-R, *lcpB*-down-F/*lcpB*-down-R sets of primers (Supplementary Table 2). The upstream and downstream regions of each gene were ligated to form an up-down fragment. The resultant fragment was digested with restriction enzymes and then cloned into pBTs. The resulting plasmids, pBTs-*lcpB*, were first electroporated into *S. aureus* strain RN4220 for modification and subsequently transformed into *S. aureus* strain ST59. The allelic replacement mutants were selected using a previously described method and were further confirmed by PCR and sequencing. N315 and Newman knockout strains were constructed in the same way.

### Complementation of Mutants

For complementation, the plasmid pLI50 was used to construct pLI50-*lcpB* complemented plasmid. First, amplified the fragment carrying the ORF and its native promoter from ST59 genomic DNA. and digested with restriction enzymes and cloned into the shuttle plasmid pLI50 to derive the plasmid pLI50-*lcpB*. The recombinant plasmid was transformed into *S. aureus* RN4220 by electroporation and then into the ST59 *lcpB* mutant to derive the complemented strain. The wild-type, *lcpB* mutant strains were transformed with the plasmid pLI50 as the control strains, resulting in the ST59-pLI50, and Δ*lcpB*-pLI50 strains respectively. The complementation strains were selected using the same method described above and were further confirmed by PCR and sequencing.

### Triton X-100-Induced Autolysis Assay

Triton X-100-stimulated autolysis was measured as described previously (Liu et al., 2016). Briefly, overnight-grown bacterial cells were diluted with TSB to 0.05 at the absorbance 600 and then allowed to grow to the early exponential phase (OD_600_ ≤ 0.8) at 37°C with shaking at 220 rpm. Cells were harvested, washed three times with PBS, resuspended in the original volume of Tris-HCl (0.05 M; pH 7.5) containing 0.05% (v/v) Triton X-100, incubated at 37°C with shaking. The autolysis level was checked by measuring the progressive decrease in absorbance (OD_600_) each hour using a microplate reader (Elx800; Bio-Tek) and analyzed by comparing the percentage of reduction. The experiment was repeated at least three times, with similar results.

### Transmission Electron Microscopy

To detect morphological changes, strains were cultivated in TSB adding 15 μg/mL chloramphenicol and allowed to grow to the early exponential phase (OD_600_ = 0.5) at 37°C with shaking at 220 rpm. Samples were prepared as previously described (Wang and Sun, 2021) and sent to the Core Facility Center for Life Science (USTC, China). Specimens were examined with a transmission electron microscopy which is operated at an accelerating voltage of 120 kV.

### Antibiotic Susceptibility Assay

Antibiotic susceptibility testing was performed by the broth microdilution method, as described previously (Wang and Sun, 2021).

### Total RNA Extraction, cDNA Generation, and Real-Time Quantitative Reverse Transcription-PCR

Normally, the overnight cultures of *S. aureus* were diluted 1:100 in TSB with 15 μg/mL chloramphenicol, grown to the early exponential (OD_600_ = 0.5), and collected. When the stress response of *S. aureus* to antibiotics was detected, 10-fold MIC level were added at the early exponential (OD_600_ = 0.5) and then cultured for 30 min. The collected cells were processed with 1 mL of RNAiso Plus (TaKaRa) in combination with 0.1-mm-diameter-silica beads in a FastPrep-24 automated system (MP biomedicals Solon, OH, United States), and then used RNase-free DNase I (TaKaRa) to remove the residual DNA. The concentration of total RNA was adjusted to 200 ng/μL. Reverse transcription was carried out with the PrimeScript 1st Strand cDNA synthesis kit (Takara) and real-time quantitative reverse transcription-PCR (RT-qPCR) was performed with SYBR Premix Ex Taq (TaKaRa) using a StepOne realtime system (Applied Biosystems). The relative quantity of cDNA measured by real-time PCR was normalized to the average abundance of wild-type strain samples using housekeeping gene *hu* as the reference gene (Valihrach and Demnerova, 2012).

### Localization of LcpB

To detect the cellular localization of LcpB, we fused LcpB with GFP at its C-terminal tail with its promoter using pALC, and FM4-64 was used to determine the location of cell membrane. For confocal microscopy, an inverted confocal laser scanning microscope (FV1000, Olympus) was used. For the observation of fluorescent signals of GFP, an argon ion laser (Ex = 488 nm, Em = 515–530 nm) was used. For the observation of FM4-64, fluorescent signals were acquired using a He-Ne laser (Ex = 559 nm, Em = 570–670 nm). Finally, all the confocal images were captured with FV10-ASW 4.2 Viewer software (Olympus).

### Expression and Purification of ΔTM-LcpB

The Gluathion S-transferases (GST)-tagged LcpB was expressed and purified using standard procedures. The fragment of the extracellular region *lcpB* ORF (30–408 residues) was amplified by PCR with the primer pair ΔTM-*lcpB*-F/ΔTM-*lcpB*-R from *S. aureus* strain ST59 genomic DNA, cloned into the expression vector pGEX-4T-2 to generate the plasmid pGEX-ΔTM-LcpB, and transformed into *E. coli* BL21 (DE3). The transformant was grown in LB at 37°C to an OD_600_ of 0.6 and induced with 0.5 mM isopropyl-β-D-1-thiogalactopyranoside (IPTG) at 16°C for additional 12 h. The cells were harvested and lysed by sonication in a lysis buffer (50 mM Tris-HCl, pH 8.0). The bound protein was eluted with an elution buffer (10 mM reduced glutathione, 50 mM Tris-HCl, pH 8.0). The purity of the protein was analyzed using SDS-PAGE, and the protein concentration was determined using the Bradford Assay Kit (Beyotime).

### Pyrophosphatase Assay

The pyrophosphatase activity of ΔTM-LcpB was determined according to a previously published protocol (Siegel et al., 2019). ΔTM-LcpB, single mutated protein, and GST (4 μg) was incubated with 2 μmol FPP Farnesyl pyrophosphate (FPP) in 50 mM Tris-HCl (pH 8.0) for 1 h at 30°C. Inorganic phosphate released from these reactions was detected by a Phosphate Assay Kit (ab270004; abcam) according to the manufacturer’s instructions. Phosphate signal was measured with a microplate reader (Elx800; Bio-Tek) at the wavelength in 600 nm. Phosphate standards were used to generate a standard curve, with samples without phosphate used as control. The phosphate concentrations in the test samples were determined by linear regression analysis of the standard curve. The results were presented as an average from three independent experiments.

### Extraction and Quantification of *Staphylococcus aureus* Wall Teichoic Acid

Overnight-grown bacterial cells were diluted with TSB to 0.05 at the absorbance 600 and then allowed to grow to the early exponential phase (OD_600_ = 0.5). Harvested 200 mL cultures of the *S. aureus* ST59 strain and extracted WTA. The extraction and analysis method strictly followed the protocol of the work of Covas et al. (2016). The quantification of WTA was applied by the measure of the phosphate group from WTA using the Phosphate Assay Kit with a microplate reader (Elx800; Bio-Tek) at the wavelength in 600 nm. The extraction of WTA were analyzed by native PAGE and detected by Alcian blue–silver staining. The results were presented as an average from three independent experiments.

### Determination of Hemolytic Activity

Hemolytic activity was determined by incubating samples with sheep red blood cells. Overnight cultures were collected by centrifugation, and supernatants (100 μL) were mixed with 900 μL phosphate-buffered saline (PBS) buffer containing 10% sheep red blood cells, and the mixtures were incubated at 37°C for proper time. It takes 1 h for ST59 strains, 2.5 h for N315 and Newman strains. The absorption of supernatant at 543 nm was measured after centrifugation. A mixture with 1 mL ddH_2_O containing 10% sheep red blood cells was used as the positive control, and a mixture with 1 mL PBS containing 10% sheep red blood cells was used as the negative control. The percentage of hemolytic activity was calculated relative to the positive control, which was regarded as 100% hemolytic activity.

### Mouse Subcutaneous Abscess Model

Outbred, immunocompetent female BALB/c mice between 5 and 6 weeks of age were purchased from Gem Pharmatech Technology Company and raised them to 6–8 weeks old. The hair on the back was removed by an animal shaver. Overnight-grown bacterial cells were diluted with TSB to 0.05 at the absorbance 600 and then allowed to grow to the early exponential phase (OD_600_ = 0.5) at 37°C with shaking at 220 rpm. Bacteria were collected, washed twice, and diluted in sterile PBS. Viable cells were counted via colony forming units (CFU) counting on TSB agar plates in order to quantify the infectious dose. Mice were inoculated with 2.5 × 10^8^ live *S. aureus* cells or PBS alone in both flanks of the back by subcutaneous injection. Abscess areas, assessed as the maximal length times width of the developing ulcers, were measured daily. Seven days after infection, the mice were sacrificed. The skin lesions were excised and homogenized in PBS. The number of CFU recovered from each individual lesion was counted by serial dilution and plated onto LB agar plates. For histopathological analyses, the skin lesions were placed in 10% formalin. Paraffin embedding and hematoxylin and eosin (H&E) staining were performed by Wuhan Servicebio Technology.

### Ethics Statement

The use and care of mice in the present study followed strictly the guidelines adopted by the Ministry of Health of the People’s Republic of China in June 2004. The protocol was approved by the Institutional Animal Care and Use Committee of the University of Science and Technology of China (USTCACUC182301015).

### Statistical Analyses

Statistical analysis was performed using Origin 2019 and GraphPad Prism 5. Data were analyzed using unpaired *t*-tests to compare two different conditions and analysis of variance for more conditions. All error bars show the standard errors of the means (SEM). All experiments were performed in biological triplicates.

## Results

### The LcpB Mutant Strain Shows Cell Wall Defects

**FIGURE 1:**
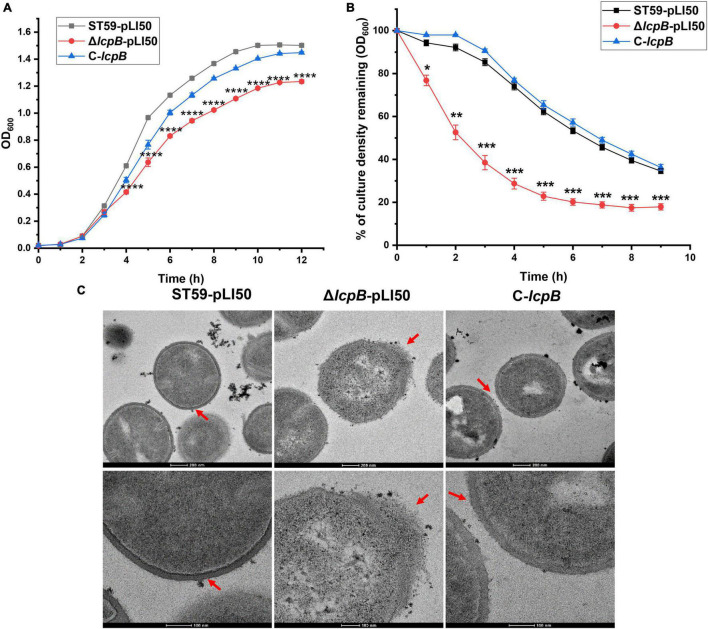
Deficiency of *lcpB* causes an abnormal cell wall morphology. **(A)** Growth of the wild-type, *lcpB* mutant, and *lcpB* complemented strains. The results were obtained from three independent experiments performed in triplicates. Data is presented as the mean ± SD or SEM. Two-tailed Student’s *t*-test is used for the comparison of statistical significance. The wild-type strain was used as the reference. *p < 0.05; **p < 0.01; ***p < 0.001, ****p < 0.0001. **(B)** Analysis of the autolysis rate. The rate of autolysis in the wild-type, *lcpB* mutant, and complemented strains was determined at 37°C using Tris-HCl buffer containing 0.05% Triton X-100. Changes in optical density were then measured upon exposure to the detergent. The results were obtained from three independent experiments performed in triplicates. Data is presented as the mean ± SD or SEM. Two-tailed Student’s *t*-test is used for the comparison of statistical significance. The wild-type strain was used as the reference. *p < 0.05; **p < 0.01; ***p < 0.001. **(C)** Transmission electron microscopy was performed to evaluate cell wall morphology. These results were obtained from three independent experiments performed in triplicates.

LcpB is considered to be a transmembrane protein that plays an important role in maintaining cell morphology in *S. aureus* (Chan et al., 2013). Therefore, in order to get a better understanding of the effects of LcpB, we knocked out *lcpB* in the clinic isolates *S. aureus* ST59 (NCBI number: CP076823, Supplementary Table 3) and constructed the complemented strain. The function of LcpB was then assessed by examining several phenotypes related to the cell wall, including change in growth, rate of autolysis, and levels of MIC (Supplementary Table 4). Notably, we first evaluated the growth of the wild-type, *lcpB* mutant, and complemented strains. The findings showed that there was a decrease in the growth rate of the *lcpB* mutant strain (Figure 1A). Thereafter, the autolytic activity of these three strains was examined. The results revealed that there was an increase in the rate of autolysis rate in the *lcpB* mutant, compared to the wild-type. The phenotype of the complemented strain was, however, same as that of the wild-type (Figure 1B). Given that autolysis is related to cell wall synthesis (Templin et al., 1999; Wang and Sun, 2021; Zamakhaeva et al., 2021), these results suggested that deficiency of *lcpB* can affect cell wall synthesis. Therefore, we used transmission electron microscopy to observe the morphology of the cell wall in these three strains. As expected, the results clearly showed that wild-type bacteria revealed regular-shaped cells, while the *lcpB* mutant strain generated deformed cells with irregular envelopes in the early stage of growth (Figure 1C middle) while the wild-type and complemented strains were regular-shaped with smooth cell walls (Figure 1C left and right). These findings further revealed that the absence of LcpB resulted in defects in the cell wall.

### LcpB Has Pyrophosphatase Activity and Affects Wall Teichoic Acid Synthesis

**FIGURE 2:**
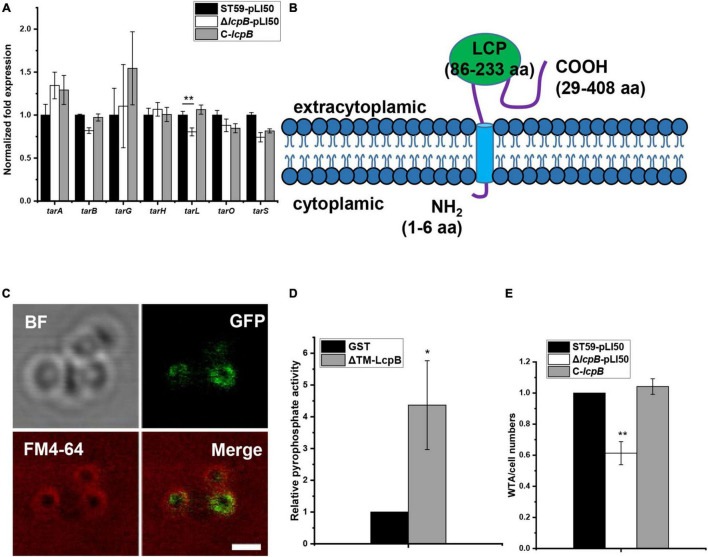
LcpB has pyrophosphatase activity and affects WTA synthesis. **(A)** The transcription levels of genes related to cell wall synthesis in the wild-type, *lcpB* mutant, and complemented strains were evaluated through RT-qPCR. The means and standard deviations were then calculated. Data is presented as the mean ± standard deviations. Two-tailed Student’s *t*-test is used for the comparison of statistical significance. The wild-type strain was used as the reference. *p < 0.05; **p < 0.01. **(B)** The putative structure of LcpB. **(C)** LcpB-GFP localized to the cell membrane. LcpB, labeled by GFP, was co-localized with the cell membrane, indicated by FM4-64. The scale bar is 1 μm. **(D)** The pyrophosphatase activity of ΔTM-LcpB. Truncated LcpB (4 μg) purified using a GST tag was incubated with 2 μmol FPP for 1 h at 30°C and GST with FPP was used as a control. The results were obtained from three independent experiments performed in triplicates, after which the means and standard deviations were calculated. Data is presented as the mean ± standard deviations. Two-tailed Student’s *t*-test is used for the comparison of statistical significance. *p < 0.05. **(E)** Quantification of WTA in the wild-type, *lcpB* mutant, and complemented strains. At least three independent experiments were performed for each assay, after which the means and standard deviations were calculated. Data is presented as the mean ± standard deviations. Two-tailed Student’s *t*-test is used for the comparison of statistical significance. *p < 0.05; **p < 0.01.

As previous studies have shown, LCP proteins can bind to the promoter region of target genes to regulate their transcription (Lazarevic et al., 1992; Cieslewicz et al., 2001; Hanson et al., 2011). In order to explore the regulatory mechanism of LcpB, we first analyzed the change of gene transcription levels in the wild-type and *lcpB* mutant strains using RNA-seq. Interestingly, the results showed that deficiency of *lcpB* had minimal effect on the transcription of genes related to cell wall synthesis or degradation (Supplementary Table 5). We next verified the results of RNA-seq. RT-qPCR results showed no significant changes in the expression of cell wall related genes (Supplementary Figure 1), including autolysin genes *lytM* and *atl* (Sugai et al., 1995; Singh et al., 2010), cell wall synthesis related genes *pbps*, *femX* (Schneider et al., 2004; Sobral and Tomasz, 2019), and cell wall hydrolysis gene *fmtA* (Komatsuzawa et al., 1997). Since LcpB is involved in WTA synthesis (Chan et al., 2013; Srisuknimit et al., 2017), we further assessed the transcription levels of genes associated with the process, including *tarG*, *tarH*, *tarO*, *tarL*, *tarS*, *tarA*, and *tarB*. The results revealed little to no differences among the wild-type, *lcpB* mutant, and complemented strains (Figure 2A). These findings suggested that LcpB may not be a transcription regulator that affects the transcription of cell wall related genes, but to be involved in cell wall synthesis.

Moreover, members of the LCP family are putative transmembrane proteins (Hubscher et al., 2008). Therefore, we assessed transmembrane segment using TMHMM, a tool that predicts the topology of proteins (Krogh et al., 2001). Analysis of the LcpB amino acid sequence suggested the existence of a short intracellular part (1–6 residues), a single transmembrane region at the N-terminal region of the protein (7–28 residues), and that the main part of LcpB was located outside the cell (29–408 residues, Figure 2B). In order to further confirm the subcellular localization of the protein, we fused it with GFP at its C-terminal tail and the fusion gene was controlled by the native promoter of *lcpB*. Confocal microscopy observation showed that LcpB exhibites plasma membrane localization which is indicated by FM4-64 (Figure 2C). These results confirmed that LcpB is indeed a transmembrane protein.

Existing literature suggests that LCP proteins may have pyrophosphatase activity (Siegel et al., 2019). Therefore, in order to verify whether LcpB is a pyrophosphatase, we attempted to express the full-length protein but the efforts were unsuccessful. Consequently, the protein was truncated and its extracellular region was expressed (ΔTM-LcpB, 30–408 residues). It is also important to note that the members of the LCP family studied to date possess pyrophosphatase activity and can hydrolyze diphosphate phosphoanhydride bonds (Siegel et al., 2019). In order to ascertain whether LcpB has the same function, a diphosphate mimetic substrate was used and farnesyl pyrophosphate (FPP) was incubated with ΔTM-LcpB, which was expressed in and purified from *Escherichia coli* (Supplementary Figure 2). The pyrophosphatase activity of the ΔTM-LcpB proteins was then determined by quantitatively measuring the amount of inorganic phosphate (Pi) released from FPP. Compared to glutathione S-transferase (GST), the negative control, there was a significant increase in the amount of phosphate produced, after incubation with FPP (Figure 2D). These results confirmed that LcpB is a pyrophosphatase. Moreover, previous research showed that mutants lacking the LCP family can release cell wall teichoic acids into the extracellular medium (Chan et al., 2013). Therefore, we assessed the level of WTA content in the wild-type, *lcpB* mutant and complemented strains, in early growth stage. The findings revealed that there was a decrease in the amount of WTA in the cell wall of the *lcpB* mutant strain (Figure 2E and Supplementary Figure 3). These results suggested that LcpB does not regulate cell wall synthesis as a transcription factor and it may affect WTA synthesis through its pyrophosphatase activity.

### Contribution of the LcpB Arginine Site in the LytR-CpsA-Psr Domain to Pyrophosphatase Activity and Wall Teichoic Acid Synthesis

**FIGURE 3:**
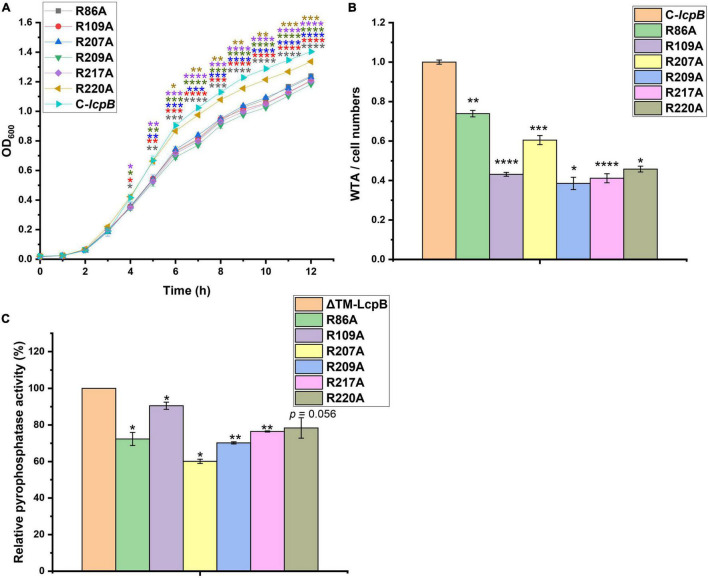
Contributions of LcpB arginine sites in the LCP domain to pyrophosphatase activity and WTA synthesis. **(A)** Growth of strains with single arginine mutations in the LCP domain and non-mutant complemented strains. The results were obtained from three independent experiments performed in triplicates. Data is presented as mean ± standard deviation. Two-tailed Student’s *t*-test is used for the comparison of statistical significance. *p < 0.05; **p < 0.01; ***p < 0.001; ****p < 0.0001. **(B)** Quantification of WTA in strains with single arginine mutation in the LCP domain and non-mutant complemented strains. At least three independent experiments were performed for each assay, after which the means and standard deviations were calculated. Data is presented as mean ± standard deviation. Two-tailed Student’s *t*-test is used for the comparison of statistical significance. *p < 0.05; **p < 0.01; ***p < 0.001; ****p < 0.0001. **(C)** Comparison of the pyrophosphatase activity of ΔTM-LcpB and ΔTM-LcpB with a single arginine mutation. Four microgram protein was incubated with 2 μmol FPP for 1 h at 30°C. The results were obtained from three independent experiments performed in triplicates, after which the means and standard deviations were calculated. Data is presented as the mean ± standard deviation. Two-tailed Student’s *t*-test is used for the comparison of statistical significance. *p < 0.05; **p < 0.01.

Members of the LCP family contain conserved arginine residues which are thought to play a key role in enzyme activity (Kawai et al., 2011; Siegel et al., 2019). In addition, there are 15 arginine sites in LcpB, all of which are located in the extracellular region (Supplementary Figure 4A). In order to determine which arginine site was key site to the pyrophosphatase activity of LcpB, we constructed single-mutated complemented strains in which these arginines were mutated to alanines. The growth rate was then tested for preliminary screening. The results showed that, compared to the non-mutated strain, arginine mutations at position 86, 109, 207, 209, 217, and 220 led to a decrease in growth rate (Figure 3A). However, the other mutations had not effect on growth (Supplementary Figure 4B). Next, we assessed the amount of WTA in these six mutants. As expected, the amount of WTA in the six mutants decreased by 40–65%, compared to the non-mutated complementary strain (Figure 3B). These results therefore indicated that these six arginine sites are important for the activity of LcpB. The findings also showed that these six arginine sites were all located in the putative LCP domain. This suggested that the LCP domain plays a crucial role in the activity of LcpB. Consequently, these six proteins with single arginine mutations were expressed (Supplementary Figure 2), after which we evaluated their pyrophosphatase activity. Corresponding to results on the amount of WTA, ΔTM-LcpB with a single arginine mutation had a decrease in pyrophosphatase activity by 70–90% (Figure 3C). These results suggested that LcpB affected the synthesis of WTA through pyrophosphatase activity and the key arginine sites in the LCP domain regulated its enzyme activity.

### Contribution of LcpB to Virulence

**FIGURE 4:**
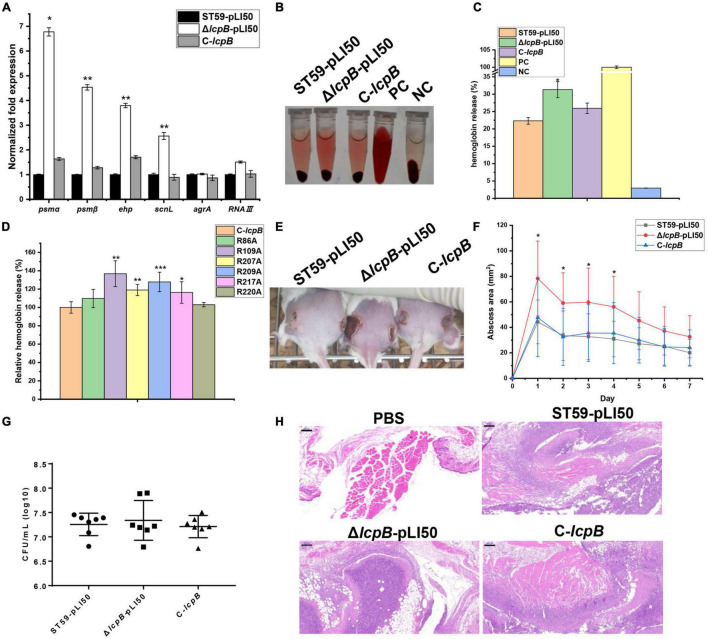
Contribution of LcpB to virulence. **(A)** The transcriptional levels of genes related to virulence in the wild-type, *lcpB* mutant, and complemented strains were examined through RT-qPCR. The means and standard deviations were then calculated and data is presented as the mean ± standard deviation. *p < 0.05; **p < 0.01. **(B)** Hemolytic activity in the wild-type, *lcpB* mutant, and complemented strains was determined by incubating samples with 10% sheep red blood cells for 1 h. PBS and ddH_2_O were used as the negative control and the positive control, respectively. **(C)** Hemolytic activity in the wild-type, *lcpB* mutant, and complemented strains was determined by measuring the absorption of supernatants at 543 nm. Data is presented as the mean ± standard deviation. Two-tailed Student’s *t*-test is used for the comparison of statistical significance. *p < 0.05. **(D)** Hemolytic activity in the *lcpB* complemented and single mutated strains was determined by measuring the absorption of supernatants at 543 nm for 1 h. Data is presented as the mean ± standard deviation. Two-tailed Student’s *t*-test is used for the comparison of statistical significance. *p < 0.05; **p < 0.01; ***p < 0.001. PBS and ddH_2_O were used as the negative control and the positive control, respectively. **(E–H)** LcpB contributes to the virulence of *S. aureus* in a mouse model of subcutaneous abscess. The mice were treated with 50 μL of PBS containing 2.5 × 10^8^ CFU of the wild-type, *lcpB* mutant, and complemented strains, or PBS alone as the control. The treatment was administered in both flanks of the back by subcutaneous injection. **(E,F)** (*n* = 6–8) The abscess area was measured daily using a caliper **(F)**. The photographic images **(E)** of representative abscesses in mice 7 days after infection. The error bars indicate the standard errors of the means, obtained from three biological replicates. Data is presented as the mean ± standard deviation. Two-tailed Student’s *t*-test is used for the comparison of statistical significance. *p < 0.05; **p < 0.01; ***p < 0.001. **(F)** CFU recovered from each abscess harvested 7 days after infection were determined through serial dilution and plating on LB agar plates. **(H)** Representative images of histological analysis (H&E stain). The scale bar is 200 μm.

We further analyzed and compared the RNA-seq data of the wild-type and *lcpB* mutant strains. The findings showed that several virulence-related genes were up-regulated (Supplementary Table 5), suggesting that LcpB may be involved in regulating virulence. In order to explore the relationship between LcpB and virulence, the RNA-seq results were first verified. Analysis through RT-qPCR showed that *psm*α, *psm*β, *scnL*, and *ehp* were significantly up-regulated (Figure 4A). However, the transcription of *agrA* and *RNAIII* showed no visible alteration, suggesting that the regulation of virulence by LcpB may be *agr*-independent. Notably, PSMs are well-characterized toxins that play a significant role in skin infection by *S. aureus* (Kennedy et al., 2010). Moreover, SCN (SCIN) is an efficient modulator of neutrophil chemotaxis, phagocytosis, and killing, whose early expression is necessary for efficient modulation of early immune responses (Rooijakkers et al., 2006). Protein encoded by *orf1855* is predicted to be a staphylococcal complement inhibitor which has high homology with SCN, and belongs to the SCN family. Therefore, the present study named *orf1855* as *scnL*. Additionally, Ehp is a secreted protein which can bind to C3 to inhibit the alternative complement activation pathway (Hammel et al., 2007; Jongerius et al., 2007). Based on the RT-qPCR results, we assessed the interaction between the bacteria and the host through the levels of hemolytic activity, and the ability of form abscesses in the wild-type, *lcpB* mutant, and complemented strains. The hemolytic assay was firstly performed using the sheep red blood cells and the percentage of hemolytic activity was calculated relative to the positive control (100% hemolytic activity) by measuring the optical density at 543 nm. The *lcpB* mutant strain displayed increased hemolytic activity, compared to the wild-type strain after incubation for 2 h at 37°C and these changes could be restored by the complemented strain (Figures 4B,C). Since our above study showed that LcpB had six key arginine sites, we also tested the hemolytic ability of these single point mutant strains. The results showed that compared to the non-mutated strain, arginine mutations at position 109, 207, 209, and 217 led to an increase in hemolytic activity (Figure 4D). These results suggest that LcpB may regulate virulence through pyrophosphatase activity. In addition, we used a mouse model of subcutaneous abscess to investigate the contribution of LcpB to the pathogenicity of *S. aureus*. The findings showed that the ability of the *lcpB* mutant strain to cause skin abscesses in mice was significantly enhanced, compared to the wild-type and *lcpB* complemented strains. This was further demonstrated by photographs of the skin lesions (Figures 4E,F). Next, we examined bacterial colonization of the skin lesions. The results revealed that these three strains had a similar level of colonization (Figure 4G). It was therefore speculated that the increase in abscess area was not caused by the difference in bacterial quantity but by the enhanced virulence of the *lcpB* mutant strains. Moreover, histological examination of the *lcpB* mutant showed more extensive inflammation with leukocyte infiltration, destruction of the skin structure (Figure 4H). Overall, these findings showed that LcpB can promote the expression of virulence genes and affect the pathogenicity of *S. aureus*.

### Regulation of LcpB Is Strain-Specific and *Agr*-Independent

**FIGURE 5:**
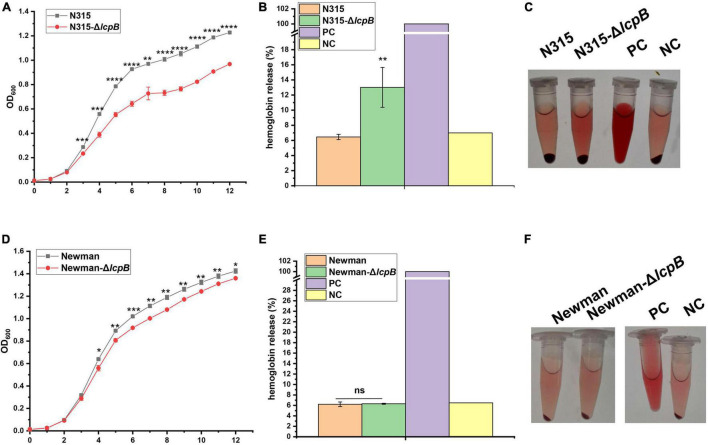
Regulation of LcpB is strain-specific and *agr*-independent. **(A)** Growth of the N315 wild-type and *lcpB* mutant strains. The results were obtained from three independent experiments performed in triplicates. Data is presented as the mean ± SD or SEM. Two-tailed Student’s *t*-test is used for the comparison of statistical significance. The wild-type strain was used as the reference. *p < 0.05; **p < 0.01; ***p < 0.001, ****p < 0.0001. **(B)** Hemolytic activity in the N315 wild-type and *lcpB* mutant strains was determined at 543 nm by incubating samples with 10% sheep red blood cells for 2.5 h. PBS and ddH_2_O were used as the negative control and the positive control, respectively. Data is presented as the mean ± standard deviation. Two-tailed Student’s *t*-test is used for the comparison of statistical significance. *p < 0.05, **p < 0.01. **(C)** Photograph for hemolytic activity in the N315 wild-type and *lcpB* mutant strains. **(D)** Growth of the Newman wild-type and *lcpB* mutant strains. The results were obtained from three independent experiments performed in triplicates. Data is presented as the mean ± SD or SEM. Two-tailed Student’s *t*-test is used for the comparison of statistical significance. The wild-type strain was used as the reference. *p < 0.05; **p < 0.01; ***p < 0.001. **(E)** Hemolytic activity in the Newman wild-type and *lcpB* mutant strains was determined at 543 nm by incubating samples with 10% sheep red blood cells for 2.5 h. PBS and ddH_2_O were used as the negative control and the positive control, respectively. Data is presented as the mean ± standard deviation. Two-tailed Student’s *t*-test is used for the comparison of statistical significance. ns, no significance. **(F)** Photograph for hemolytic activity in the Newman wild-type and *lcpB* mutant strains.

Wanner et al. (2017) reported before that *agr* system participates in WTA synthesis and virulence through regulating *tarH*. Our study showed that *lcpB* knockout affected WTA synthesis and virulence, but the transcriptional level of *agrA*, *RNAIII*, and *tarH* showed no significant difference. In order to examine whether the regulation of virulence by LcpB is *agr*-independent or not, we tried to knock out *agr* system in ST59, but failed. Therefore, another MRSA strain N315, an *agr* system deficient strain, was used for further study. Interestingly, *lcpB* deficiency greatly slowed down the growth (Figure 5A) and enhanced the hemolytic activity (Figures 5B,C). This is consistent with the phenotype of ST59 strain. This suggested that the regulation of WTA synthesis and virulence by LcpB is *agr*-independent.

In order to examine whether the mechanism of LcpB is common in *Staphylococcus aureus* or not, we knocked out *lcpB* in MSSA strain Newman. The results showed that *lcpB* deletion slightly affected the growth rate (Figure 5D) and had no visible alteration on hemolysis (Figures 5E,F). These results suggest that the effect of LcpB was strain specific and it may play a more important role in MRSA strains.

## Discussion

The present study demonstrates the significant role of LcpB, an important member of the LCP family, in clinical isolates of *S. aureus* ST59. The results showed that LcpB is a transmembrane protein with pyrophosphatase activity that not only affects the synthesis of WTA but also regulates bacterial growth, autolysis, and maintenance of cell wall morphology. Further experiments also showed that there are six key arginine sites in LcpB that affect enzyme activity and all of them are located in the putative LCP domain. In addition, the findings revealed that LcpB plays a role in regulating virulence. The results specifically showed that LcpB is involved in the transcriptional regulation of virulence genes and affects many physiological processes such as hemolytic activity and abscess formation in an *agr*-independent manner. In addition, the study also found that the role of LcpB was strain-specific. Our study therefore uncovers the significant role of LcpB in both cell wall synthesis and regulation of virulence.

Members of the LytR-CpsA-Psr family are putative transmembrane proteins that have a common structure consisting of a short N-terminal cytoplasmic domain, a transmembrane region, and an LCP domain that was predicted to be extracellular (Hubscher et al., 2008). LCP proteins are thought to be involved in many physiological processes (Amer and Clubb, 2014; Baumgart et al., 2016). Additionally, previous studies revealed that LCP proteins can bind to the promoter region of target genes to regulate their transcription (Lazarevic et al., 1992; Cieslewicz et al., 2001; Hanson et al., 2011). Recent, research also confirmed that proteins in the LCP family have pyrophosphatase or phosphotransferase activities and can participate in WTA synthesis (Harrison et al., 2016; Srisuknimit et al., 2017; Siegel et al., 2019). Meanwhile, structural prediction suggested that LcpB was not a transcription factor. Moreover, analysis through RNA-seq and RT-qPCR showed that deficiency of *lcpB* had no significant effect on the transcription of genes related to cell wall synthesis. In addition, LcpB was shown to have pyrophosphatase activity, which affected WTA synthesis. This study therefore supports the idea that LCP proteins are directly involved in WTA synthesis. In addition, analysis of the ability of LcpB to regulate virulence revealed that several virulence genes were up-regulated, including *ehp*, *scnL*, *psm*α, and *psm*β. There was, however, no decrease in the expression levels of *agr* and *RNAIII* in the *lcpB* knockout strain whose concentration of WTA had decreased (Figure 2A), contrary to the findings by Wanner et al. (2017). However, our study showed that the regulation of virulence by LcpB was *agr*-independent. This suggests that there may be multiple regulatory pathways involved in the interaction between WTA synthesis and virulence, and the specific mechanism needs to be explored further.

In summary, our study showed that LcpB is a pyrophosphatase, which can regulate both WTA synthesis and virulence in *S. aureus* ST59. And this regulation of LcpB is *agr*-independent and strain-specific. Recent research on the development of antimicrobial substances identified WTA as an ideal target for novel anti-infective strategies and antibiotics. The present study will therefore aid in the development of novel anti-staphylococcal strategies that can especially be helpful in combating MRSA.

## Data Availability Statement

The original contributions presented in the study are included in the article/Supplementary Material, further inquiries can be directed to the corresponding author/s.

## Ethics Statement

The protocol was approved by the Institutional Animal Care and Use Committee of the University of Science and Technology of China (USTCACUC182301015).

## Author Contributions

TP designed the study, acquired, analyzed and interpreted the data, and prepared the manuscript. JG conceived the study and acquired the data. YL analyzed, interpreted the data, and revised the manuscript. BS and YL supervised the project and obtained funding. All authors discussed the data and read the manuscript.

## Conflict of Interest

The authors declare that the research was conducted in the absence of any commercial or financial relationships that could be construed as a potential conflict of interest.

## Publisher’s Note

All claims expressed in this article are solely those of the authors and do not necessarily represent those of their affiliated organizations, or those of the publisher, the editors and the reviewers. Any product that may be evaluated in this article, or claim that may be made by its manufacturer, is not guaranteed or endorsed by the publisher.

## Supplementary Material

Supplementary Figure 1 The transcriptional levels of genes related to cell wall synthesis showed little to no differences among the wild-type, *lcpB* mutant, and complemented strains. The transcriptional levels were evaluated through RT-qPCR. The means and standard deviations were then calculated. Data is presented as the mean ± standard deviations. **p* < 0.05.

Supplementary Figure 2 The expression of GST, ΔTM-LcpB, and mutated LcpB.

Supplementary Figure 3 The extraction of WTA. WTA of the wild-type, *lcpB* mutant, and complemented strains was analyzed by native PAGE and detected by Alcian blue–silver staining.

Supplementary Figure 4 The distribution of arginine in LcpB and the mutation sites of arginine that did not affect the growth. **(A)** The distribution of arginine in LcpB. **(B)** Growth of strains with single arginine mutations that did not affect the growth and non-mutant complemented strains. The results were obtained from three independent experiments performed in triplicates.
