# Corrigendum: LcpB is a pyrophosphatase responsible for wall teichoic acid synthesis and virulence in *Staphylococcus aureus* clinical isolate ST59

**DOI:** 10.3389/fmicb.2023.1229396

**Published:** 2023-07-04

**Authors:** Ting Pan, Jing Guan, Yujie Li, Baolin Sun

**Affiliations:** ^1^Department of Oncology, The First Affiliated Hospital of USTC, Division of Life Sciences and Medicine, University of Science and Technology of China, Hefei, Anhui, China; ^2^Department of Gastroenterology, The First Affiliated Hospital of Anhui Medical University, Hefei, China

**Keywords:** LCP family, pyrophosphatese, *Staphylococcus aureus*, virulence, wall teichoic acid synthesis, *agr* system

In the published article, there was an error in the affiliation section [1, 2, 3].

Instead of “[1 Department of Oncology, The First Affiliated Hospital, University of Science and Technology of China, Hefei, China, 2 Department of Life Science and Medicine, University of Science and Technology of China, Hefei, China, 3 Department of Gastroenterology, The First Affiliated Hospital of Anhui Medical University, Hefei, China]”.

The affiliations should be corrected as follows:

“[1 Department of Oncology, The First Affiliated Hospital of USTC, Division of Life Sciences and Medicine, University of Science and Technology of China, Hefei, Anhui, China, 2 Department of Gastroenterology, The First Affiliated Hospital of Anhui Medical University, Hefei, China]”.

The authors apologize for this error and state that this does not change the scientific conclusions of the article in any way. The original article has been updated.

