# Supplementary figures and images for: LcpB Is a Pyrophosphatase Responsible for Wall Teichoic Acid Synthesis and Virulence in Staphylococcus aureus Clinical Isolate ST59
Source: Front Microbiol. 2021 Dec 16;12:788500. doi: 10.3389/fmicb.2021.788500 (PMC8716876; doi:10.3389/fmicb.2021.788500)

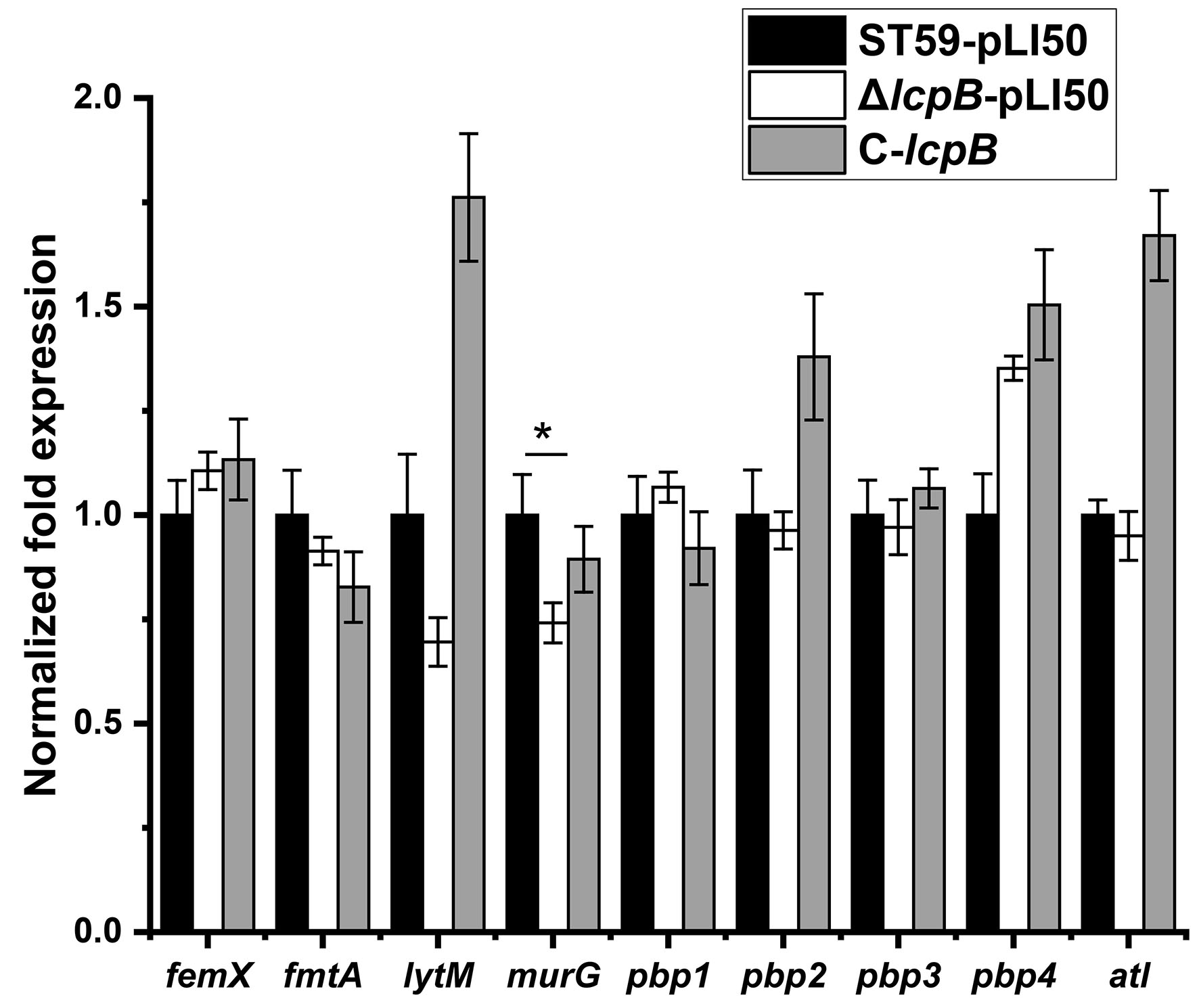

Supplement: Supplementary Figure 1 — The transcriptional levels of genes related to cell wall synthesis showed little to no differences among the wild-type, lcpB mutant, and complemented strains. The transcriptional levels were evaluated through RT-qPCR. The means and standard deviations were then calculated. Data is presented as the mean ± standard deviations. *p < 0.05. [file Image_1.JPEG]

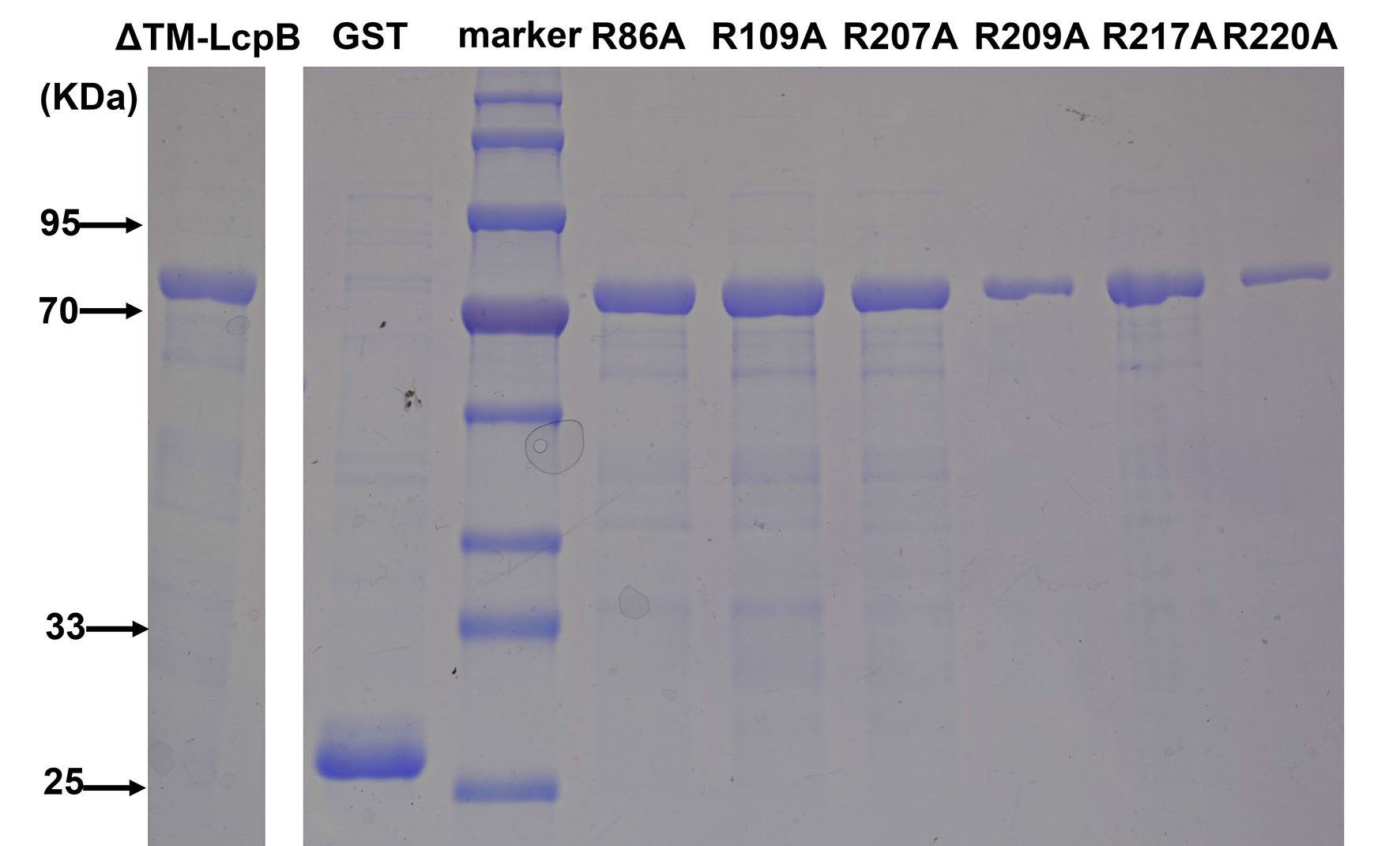

Supplement: Supplementary Figure 2 — The expression of GST, ΔTM-LcpB, and mutated LcpB. [file Image_2.JPEG]

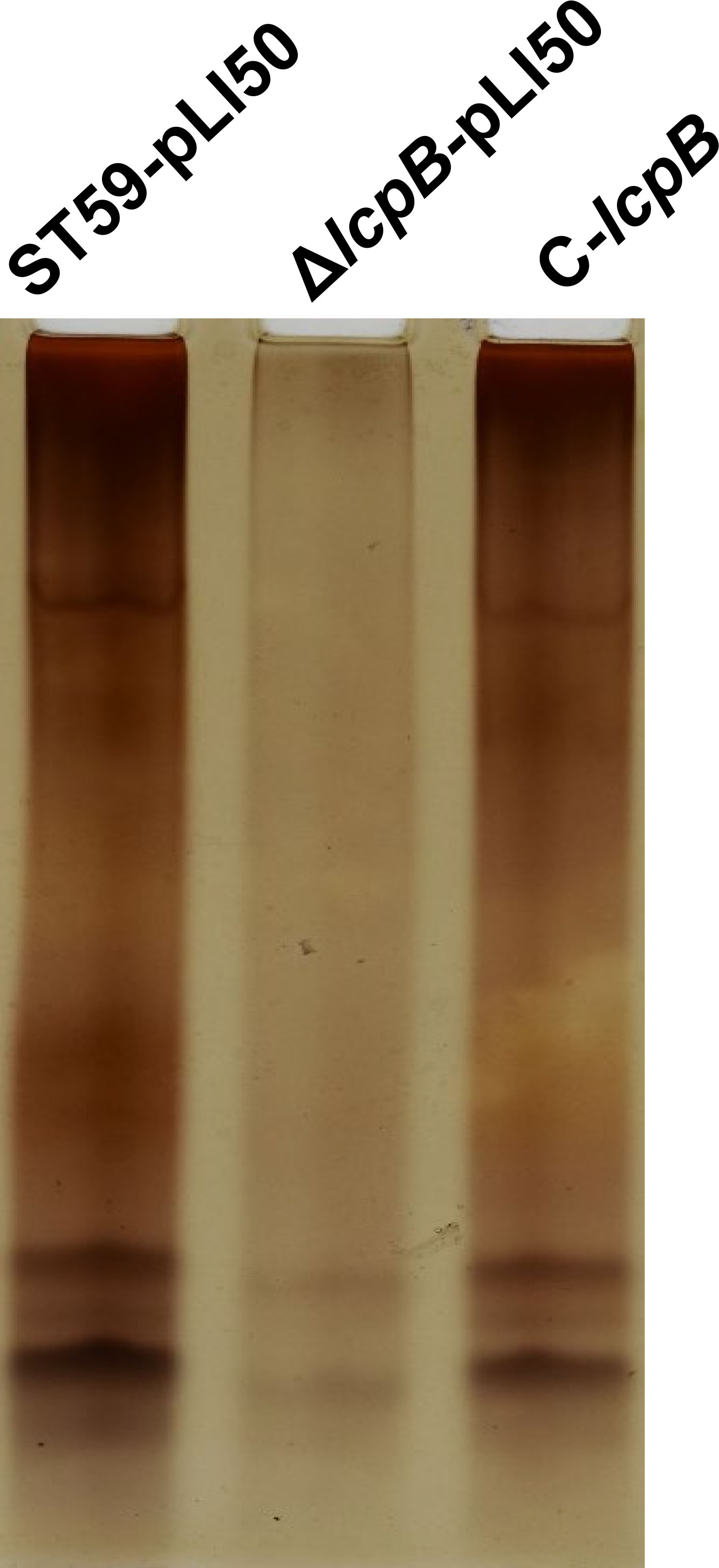

Supplement: Supplementary Figure 3 — The extraction of WTA. WTA of the wild-type, lcpB mutant, and complemented strains was analyzed by native PAGE and detected by Alcian blue–silver staining. [file Image_3.JPEG]

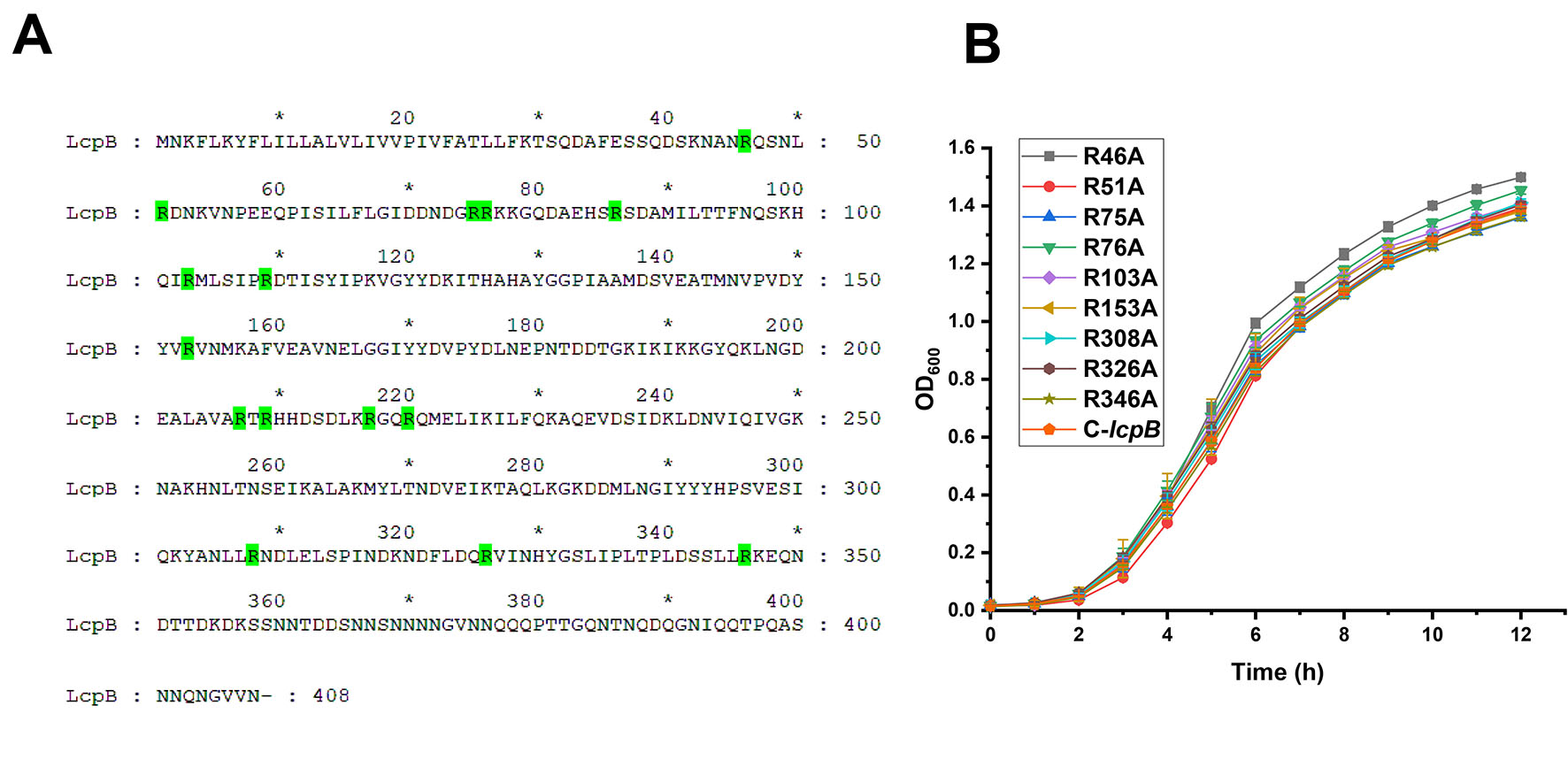

Supplement: Supplementary Figure 4 — The distribution of arginine in LcpB and the mutation sites of arginine that did not affect the growth. (A) The distribution of arginine in LcpB. (B) Growth of strains with single arginine mutations that did not affect the growth and non-mutant complemented strains. The results were obtained from three independent experiments performed in triplicates. [file Image_4.JPEG]
