# Supplementary material for: LcpB Is a Pyrophosphatase Responsible for Wall Teichoic Acid Synthesis and Virulence in Staphylococcus aureus Clinical Isolate ST59
Source: Front Microbiol. 2021 Dec 16;12:788500. doi: 10.3389/fmicb.2021.788500 (PMC8716876; doi:10.3389/fmicb.2021.788500)
Supplement: Supplementary file 5 [file Table_1.DOCX]

Table S1 Strains and plasmids used in this study

| Strain and plasmid | Relevant genotype^a^ | Source^b^ |
| --- | --- | --- |
| *S.aureus* strains |  |  |
| RN4220 | 8325-4 r^-^, initial recipient for modification of plasmids which are introduced into *S. aureus* from *E. coli* | NARSA |
| ST59 | CA-MRSA, SCC*mec* type Ⅳ, wildtype | This study |
| Δ*lcpB* | ST59 strain deletion of *lcpB* | This study |
| Δ*lcpB-*pLI50 | Δ*lcpB* with pLI50 | This study |
| Δ*lcpB-*pALC-*lcpB* | Δ*lcpB* with pALC containing ORF of *lcpB* | This study |
| C-*lcpB* | ST59 *lcpB* complemented strain with pLI50 | This study |
| C-*lcpB*-RXA | ST59 *lcpB* complemented strain with single arginine to alanine mutation | This study |
| N315 | HA-MRSA, SCC*mec* type Ⅱ, *agr*-deficient | NARSA |
| N315-Δ*lcpB* | N315 strain deletion of *lcpB* | This study |
| Newman | HA-MSSA, α- and β-toxin deficient | NARSA |
| Newman-Δ*lcpB* |  | This study |
| *E.coli* strains |  |  |
| *Trans1*-T1 | Clone host strain | TransGen |
| BL21(DE3) | Expression strain | TransGen |
|  |  |  |
| Plasmids |  |  |
| pBTs | Shuttle vector, temperature sensitive, Amp^r^ Cm^r^ | (Hu et al., 2015) |
| pBTs-Δ*vraSR* | pBTs containing upstream and downstream fragments of *vraSR*, for *vraSR* deletion, Amp^r^ Cm^r^ | This study |
| pBTs-Δ*lcpB* | pBTs containing upstream and downstream fragments of *lcpB*, for *lcpB* deletion, Amp^r^ Cm^r^ | This study |
| pLI50 | Shuttle vector, Amp^r^ Cm^r^ | (Lee et al., 1991) |
| pLI50-*vraSR* | pLI50 derivative, harboring ORF of *vraSR* and its promoter, Amp^r^ Cm^r^ | This study |
| pLI50-*lcpB* | pLI50 derivative, harboring ORF of *lcpB* and its promoter, Amp^r^ Cm^r^ | This study |
| pGEX-4T-2 | Expression vector with a GST tag, Amp^r^ | TransGen |
| pGEX-ΔTM-*lcpB* | pGEX-4T-2 derivative, with ORF of extracellular *lcpB* located at the N-terminal of GST, Amp^r^ | This study |
| pGEX-ΔTM-*lcpB* | pGEX-4T-2 derivative, with ORF of extracellular *lcpB* located at the N-terminal of GST, Amp^r^ | This study |
| pGEX-ΔTM-*lcpB*-RXA | pGEX-ΔTM-*lcpB* derivative, with single arginine to alanine mutation, Amp^r^ | This study |
| pET28 (+) | Expression vector with a hexa-histidine tag, Kan^r^ | Novagen |
| pET-vraR | pET28a(+) derivative, with ORF of vraR, Kan^r^ | This study |
| pALC | fluorescent vector with GFP | Novagen |
| pALC-LcpB | pALC derivative, with ORF of *lcpB* located at the C-terminal of GFP, Amp^r^ | This study |

^a^: r−, restriction system negative; Kan^r^, kanamycin resistant; Amp^r^, ampicillin resistant; Cm^r^, chloramphenicol resistant.

^b^: NARSA, Network on Antimicrobial Resistance in *Staphylococcus aureus*.

Table S2 Primers used in this study

| Primer | Oligonucleotide (5’-3’)^a^ | Application |
| --- | --- | --- |
| RT-*hu*-F | AAAAAGAAGCTGGTTCAGCAGTAG | RT-qPCR |
| RT-*hu*-R | TTTACGTGCAGCACGTTCAC | RT-qPCR |
| RT-*lcpB*-F | GTTGGCTATTACGATAAG | RT-qPCR |
| RT-*lcpB*-R | TCATATTGACTCTCACATA | RT-qPCR |
| RT-*psmα*-F | GTATCATCGCTGGCATCA | RT-qPCR |
| RT-*psmα*-R | AAGACCTCCTTTGTTTGTTATG | RT-qPCR |
| RT-*psmβ*-F | TGGACTAGCAGAAGCAATC | RT-qPCR |
| RT-*psmβ*-R | TAGTAAACCCACACCGTTAG | RT-qPCR |
| RT-*ehp*-F | GCTGCTATTAGTTTAACGGT | RT-qPCR |
| RT-*ehp*-R | GTGCTTTACGGTGTGTTG | RT-qPCR |
| RT-*scnL*-F | ATCAACAGCGATAGCAACT | RT-qPCR |
| RT-*scnL*-R | CAACTTTAGCTTCAGCCATT | RT-qPCR |
| RT-*agrA*-F | TATGGCGATTGACGACAA | RT-qPCR |
| RT-*agrA*-R | GCAGTAATTCAGTGTATGTTCA | RT-qPCR |
| RT-*RNAⅢ*-F | GGCTCACGACCATACTTAT | RT-qPCR |
| RT- *RNAⅢ*-R | CTTGGACTCAGTGCTATGT | RT-qPCR |
| RT-*lytM*-F | CCCAACAAGCACATAC | RT-qPCR |
| RT- *lytM*-R | TTGTGCATCTACGAATGT | RT-qPCR |
| RT-*femX*-F | TTATAGTAATAAAGAGGCGTTAAA | RT-qPCR |
| RT- *femX*-R | ATCCTTTATGTTTAAAACCAAG | RT-qPCR |
| RT-*fmtA*-F | CTTGATTGTTTACTTTTGAAAAC | RT-qPCR |
| RT- *fmtA*-R | TTTTGGCAATATACAGCAATT | RT-qPCR |
| RT-*murG*-F | TATTTCGAGTGGTAAATTAAGAAG | RT-qPCR |
| RT-*murG*-R | TAATGATTTGGCTGCAAT | RT-qPCR |
| RT-*pbp1*-F | TTAAGAATGCACAACAACC | RT-qPCR |
| RT-*pbp1*-R | GATAATTTCTTTGCAGTCTCT | RT-qPCR |
| RT-*pbp2*-F | ATTATGCTTGGAAAGCAC | RT-qPCR |
| RT-*pbp2*-R | AAGTACTGCGTCTTTCA | RT-qPCR |
| RT-*pbp3-*F | AATGTTGGATACGGCTGAA | RT-qPCR |
| RT-*pbp3*-R | GCAACTGAATCCAGAAATCT | RT-qPCR |
| RT-*pbp4*-F | TACAAGCAGGAAATCAATATG | RT-qPCR |
| RT-*pbp4*-R | ATGTTAAGTACATTGTCATTAATTT | RT-qPCR |
| RT-*atl*-F | GGTATTCGTGCTTCTGTT | RT-qPCR |
| RT-*atl*-R | GATGTTATGGCTTGTATTGTT | RT-qPCR |
| RT-*tagA*-F | TCATGTAAATCATCAAAAAGT | RT-qPCR |
| RT-*tagA*-R | CAGTTCAATTCGTTTCACTA | RT-qPCR |
| RT-*tagB*-F | ATGTTGTATTTTGGGCTT | RT-qPCR |
| RT-*tagB*-R | CTTTATCAATAGCCCTATTA | RT-qPCR |
| RT-*tarG*-F | ATCAGTATGTGGTTCTTCATC | RT-qPCR |
| RT-*tarG*-R | TGCTGCACGGTATGATTCAG | RT-qPCR |
| RT-*tarL*-F | TTCCATTCTACGCTGAAGCA | RT-qPCR |
| RT-*tarL*-R | TGTTGGTGCGAAAAGAATGA | RT-qPCR |
| RT-*tarO*-F | TTCCATCCTGCCAAAATA | RT-qPCR |
| RT-*tarO*-R | GAATGGAACTGCTAAGATAACA | RT-qPCR |
| RT-*tarS*-F | CGACCATTGTTACACTACG | RT-qPCR |
| RT-*tarS*-R | TGTCCTTCGCCAATCATAA | RT-qPCR |
| RT-*tarH*-F | ATCATTGGCGGTTCTTTGTC | RT-qPCR |
| RT-*tarH*-R | TGCACGCATACCACTTGAAT | RT-qPCR |
| P*lcpB*-R-biotin | GATTGATGCTCCTATATTA | EMSA |
| P*lcpB*-F | TCACTTTGTATAAATCACTCG | EMSA |
| *lcpB*-F | ACGCgtcgacTAGCTTCCTAATAATACAAC | complementation *lcpB* |
| *lcpB*-R | CCGgagctcTTCATTGTTTCGGTAATGC | complementation *lcpB* |
| C-GFP-F-LcpB-EcoRI | CCGgaattctagcttcctaataatacaac | LcpB location |
| C-GFP—LcpB-R | aagttcttctcctttaatgtttacaacaccattttggttatttgaagcttg | LcpB location |
| C-GFP-F | aaaatggtgttgtaaacattaaaggagaagaacttttcactggagt | LcpB location |
| C-GFP-R-PstI | AActgcagttatttgtagagctcatccatgccatgt | LcpB location |
| *lcpB*-up-F | CGGggtaccTCCTCTGGGACGCCTTGTTC | *lcpB* deletion |
| *lcpB*-up-R | GGAGCATCAATCTATTATATGACGATTAA | *lcpB* deletion |
| *lcpB*-down-F | TTAATCGTCATATAATAGATTGATGCTCC | *lcpB* deletion |
| *lcpB*-down-R | ACGCgtcgacCAATGGGATAGGCTTAGAT | *lcpB* deletion |
| ΔTM-*lcpB*-F | CGCggatccACGTCCCAAGATGCATTCGA | ΔTM-LcpB expression |
| ΔTM-*lcpB*-R | CGCggatccAGATCAGACGCTATGATTTTA | ΔTM-LcpB expression |
| *lcpB*-R46A-F | ATGCTAATgcTCAATCCAACTTAAGAGATAATAAAGTCAATCCCGA | point mutation of *lcpB* |
| *lcpB*-R46A-R | GTTGGATTGAgcATTAGCATTTTTGCTGTCTTGAGAAGAT | point mutation of *lcpB* |
| *lcpB*-R51A-F | AATCCAACTTAgcAGATAATAAAGTCAATCCCGAAGAACAACC | point mutation of *lcpB* |
| *lcpB*-R51A-R | TTATTATCTgcTAAGTTGGATTGACGATTAGCATTTTTG | point mutation of *lcpB* |
| *lcpB*-R75A-F | AACGATGGAgcAAGAAAAAAAGGACAGGATGCTGAACAC | point mutation of *lcpB* |
| *lcpB*-R75A-R | TTTTTTTCTTgcTCCATCGTTATCATCGATACCTAAGAAAA | point mutation of *lcpB* |
| *lcpB*-R76A-F | CGATGGAAGAgcAAAAAAAGGACAGGATGCTGAACACTCTAG | point mutation of *lcpB* |
| *lcpB*-R76A-R | TCCTTTTTTTgcTCTTCCATCGTTATCATCGATACCTAAG | point mutation of *lcpB* |
| *lcpB*-R86A-F | TGAACACTCTgcATCAGACGCTATGATTTTAACTACTTTTAATC | point mutation of *lcpB* |
| *lcpB*-R86A-R | GCGTCTGATgcAGAGTGTTCAGCATCCTGTCCTTTTTTTCTTC | point mutation of *lcpB* |
| *lcpB*-R103A-F | AACATCAAATTgcAATGCTTAGTATTCCACGTGATACTATCAG | point mutation of *lcpB* |
| *lcpB*-R103A-R | CTAAGCATTgcAATTTGATGTTTTGATTGATTAAAAGTAGTTAA | point mutation of *lcpB* |
| *lcpB*-R109A-F | TTAGTATTCCAgcTGATACTATCAGCTACATTCCTAAAGTTGGCTA | point mutation of *lcpB* |
| *lcpB*-R109A-R | ATAGTATCAgcTGGAATACTAAGCATTCTAATTTGATGTTTTG | point mutation of *lcpB* |
| *lcpB*-R153A-F | TTATTATGTGgcAGTCAATATGAAAGCATTTGTCGAGGCTGT | point mutation of *lcpB* |
| *lcpB*-R153A-R | ATATTGACTgcCACATAATAATCAACAGGTACATTCATGGTAG | point mutation of *lcpB* |
| *lcpB*-R207A-F | TAGCAGTTGCTgcAACTAGACATCATGATTCAGACTTGAAACG | point mutation of *lcpB* |
| *lcpB*-R207A-R | TGTCTAGTTgcAGCAACTGCTAAAGCCTCATCACCATTTAATT | point mutation of *lcpB* |
| *lcpB*-R209A-F | TGCTAGAACTgcACATCATGATTCAGACTTGAAACGCGGACA | point mutation of *lcpB* |
| *lcpB*-R209A-R | TCATGATGTgcAGTTCTAGCAACTGCTAAAGCCTCATCAC | point mutation of *lcpB* |
| *lcpB*-R217A-F | CAGACTTGAAAgcCGGACAACGTCAAATGGAATTAATTAAGAT | point mutation of *lcpB* |
| *lcpB*-R217A-R | CGTTGTCCGgcTTTCAAGTCTGAATCATGATGTCTAGTTC | point mutation of *lcpB* |
| *lcpB*-R220A-F | AACGCGGACAAgcTCAAATGGAATTAATTAAGATTTTATTCC | point mutation of *lcpB* |
| *lcpB*-R220A-R | TCCATTTGAgcTTGTCCGCGTTTCAAGTCTGAATCATGATGTC | point mutation of *lcpB* |
| *lcpB*-R308A-F | CTAACTTACTTgcTAATGATTTAGAATTATCACCTATCAATGA | point mutation of *lcpB* |
| *lcpB*-R308A-R | AAATCATTAgcAAGTAAGTTAGCATATTTTTGAATGCTTTCAA | point mutation of *lcpB* |
| *lcpB*-R326A-F | TCTTAGATCAAgcTGTTATCAACCATTATGGTTCATTAATACC | point mutation of *lcpB* |
| *lcpB*-R326A-R | TTGATAACAgcTTGATCTAAGAAATCATTTTTATCATTGATAG | point mutation of *lcpB* |
| *lcpB*-R346A-F | GTAGTTTATTGgcAAAAGAACAAAACGATACGACAGATAAAGATAA | point mutation of *lcpB* |
| *lcpB*-R346A-R | TGTTCTTTTgcCAATAAACTACTATCTAAAGGCGTTAATGGTA | point mutation of *lcpB* |
| N315-*lcpB*-up-F | CgagctcCGGTGTGTTTTTAATACC | *lcpB* deletion in N315 strain |
| N315-*lcpB*-up-R | GTTAATCGTCATATAATAGATTGATGCTCCTATATTAAATTT | *lcpB* deletion in N315 strain |
| N315-*lcpB*-down-F | AAATTTAATATAGGAGCATCAATCTATTATATGACGATTAACA | *lcpB* deletion in N315 strain |
| N315-*lcpB*-down-R | ACGCgtcgacGGGCGTATTCGAGCAGTGGC | *lcpB* deletion in N315 strain |
| Newman-*lcpB*-up-F | ACGCgtcgacccataacctttactcattttc | *lcpB* deletion in Newman strain |
| Newman-*lcpB*-up-R | cgtcatataatagattgatgctcctatattaaattt | *lcpB* deletion in Newman strain |
| Newman-*lcpB*-down-F | ggagcatcaatctattatatgacgattaacatttactatttatg | *lcpB* deletion in Newman strain |
| Newman-*lcpB*-down-R | Cgagctcccattatcatatccaatgag | *lcpB* deletion in Newman strain |

^a^: Lowercase letters: restriction endonuclease recognition sites or mutant sites

Table S3 Typing information of ST59

| Category | Typing |
| --- | --- |
| Multilocus sequence typing | ST59 |
| Spa typing | t437 |
| SCC*mec* typing | IV |

Table S4 MICs for different cell wall-targeting antimicrobials in *lcpB* mutants and ST59 wildtype strain

|  | MIC (mg/L) of indicated antibiotic | | | |
| --- | --- | --- | --- | --- |
| Strain | Daptomycin | Oxacillin | Teicoplanin | Vancomycin |
| ST59 | 2 | 128 | 1 | 1 |
| Δ*lcpB* | 2 | 64 | 1 | 1 |

Table S5 Gene expression analysis of *lcpB* mutants compared with ST59 wildtype strain using RNA-seq

| ORF | Gene | Product of function | Fold change^a^ |
| --- | --- | --- | --- |
| orf1034 | *ehp* | fibrinogen-binding protein | 5.590 |
| orf1056 | - | hypothetical protein | 4.605 |
| orf0655 | - | hypothetical protein | 4.205 |
| orf1883 | *hlb* | Phospholipase C | 4.188 |
| orf2277 | - | acetyltransferase | 4.088 |
| orf1885 | - | *hlgb*-like protein, gamma-hemolysin component B | 3.259 |
| orf0178 | - | hypothetical membrane protein | 3.122 |
| orf0822 | - | pyridine nucleotide-disulfide oxidoreductase | 3.063 |
| orf1035 | - | hypothetical membrane protein | 3.052 |
| orf1039 | *scnL* | SCIN family, Staphylococcal complement inhibitor, fibrinogen-binding protein | 3.043 |
| orf2355 | *sasG* | Accumulation-associated protein | 0.333 |
| orf1480 | *dnaK* | molecular chaperone DnaK | 0.311 |
| orf1656 | *ribBA* | GTP cyclohydrolase | 0.305 |
| orf1893 | yafV | Omega-amidase | 0.288 |
| orf1958 | *sceD* | transglycosylase | 0.283 |
| orf0717 | *clpP* | ATP-dependent Clp protease proteolytic subunit | 0.281 |
| orf1888 | *groL* | 60 kDa chaperonin | 0.281 |
| orf0776 | _ | hypothetical protein | 0.272 |
| orf0789 | _ | hypothetical protein | 0.270 |
| orf2215 | *hrtB* | hemin ABC transporter permease | 0.259 |
| orf0935 | _ | hypothetical protein | 0.241 |
| orf1481 | *grpE* | heat shock protein GrpE | 0.214 |
| orf2214 | *hrtA* | hemin ABC transporter ATP-binding protein | 0.175 |
| orf0936 | *lcpB* | LytR_cpsA_psr domain-containing protein | 0.005 |

a: Compared with the two biological replicates, there were 35 genes up-regulated and 62 genes down regulated, all of which changed by two times. Here, the gene whose expression difference was more than three times was listed.
